# Clinical and functional characteristics, possible causes, and impact of chronic cough in patients with cerebellar ataxia, neuropathy, and bilateral vestibular areflexia syndrome (CANVAS)

**DOI:** 10.1007/s00415-023-12001-9

**Published:** 2023-11-02

**Authors:** Esther Palones, Elena Curto, Vicente Plaza, Lidia Gonzalez-Quereda, Alba Segarra-Casas, Luis Querol, Federico Bertoletti, María José Rodriguez, Pía Gallano, Astrid Crespo-Lessmann

**Affiliations:** 1 Department of Medicine, Universitat Autònoma de Barcelona, Barcelona, Spain; 2 Department of Respiratory Medicine, Sant Pau Biomedical Research Institute (IIB SANT PAU), Hospital de la Santa Creu i Sant Pau, Barcelona, Spain; 3 Genetics Department, Institute of Biomedical Research Sant Pau (IIB SANT PAU), Hospital de la Santa Creu i Sant Pau, Barcelona, Spain; 4 Genetics and Microbiology Department, Universitat Autònoma de Barcelona, Barcelona, Spain; 5 Neuromuscular Disease Unit, Department of Neurology, Hospital de la Santa Creu i Sant Pau, Barcelona, Spain; 6 Department of Digestive Pathology, Hospital de la Santa Creu i Sant Pau, Barcelona, Spain; 7 Networked Biomedical Research Centre for Rare Diseases (CIBERER), Instituto de Salud Carlos III, Madrid, Spain

**Keywords:** Cerebellar ataxia, CANVAS, Gastroesophageal reflux, *RFC1*, Chronic cough

## Abstract

Cerebellar ataxia with neuropathy and bilateral vestibular areflexia syndrome (CANVAS) is an hereditary autosomal recessive disease. Recent studies propose including chronic cough (CC) as a symptom of CANVAS. For 10 patients with CANVAS as genetically confirmed by biallelic expansion of the AAGG repeat motif (AAGGGexp) in intron 2 of replication factor C subunit 1 (*RFC1*), our aim was, as a multidisciplinary team, to describe clinical and functional characteristics and possible causes of CC following European Respiratory Society (ERS) recommendations, and to evaluate CC impact on quality of life (QoL) using self-administered questionnaires (Cough Severity Diary, Leicester Cough Questionnaire, Discrete Emotions Questionnaire, and EQ-5D-5L). In all 10 patients, the CC was a dry cough that developed several years prior to the neurological symptoms (mean 14.2 years); 7 patients had symptoms compatible with gastroesophageal reflux (GER), 5 with pathological GER diagnosed by 24-h esophageal pH testing, and 6 patients had impaired esophageal motility diagnosed by high-resolution esophageal manometry, most frequently ineffective peristalsis. Although further studies are required for confirmation, we conclude that CC may be a characteristic prodrome of CANVAS and may be related to GER and esophageal disorders. Furthermore, CC affects patients’ QoL, especially in the psychosocial sphere.

## Introduction

Cerebellar ataxia with neuropathy and bilateral vestibular areflexia syndrome (CANVAS) is a rare neurological disorder of autosomal recessive inheritance. In 2019, for the first time, the underlying genetic basis of this syndrome was described as biallelic expansion of the AAGG repeat motif (AAGGGexp) in intron 2 of replication factor C subunit 1 (*RFC1*) [1]. It has recently been reported that, exceptionally, CANVAS may also be due to AAGGGexp expansion and a truncating variant in compound heterozygosity in the *RFC1* gene [2–4].

CANVAS is clinically characterized by combined adult-onset progressive ataxia, sensory neuropathy, and bilateral vestibular areflexia [5–7]. Also reported is a high prevalence (30–97%) of chronic cough (CC) [1, 8–11], which appears to precede the onset of neurological symptoms by decades (12–30 years) [12–14]. In fact, recent studies suggest that CC is a CANVAS-associated symptom that should guide CANVAS diagnosis if associated with at least 2 components of the main triad (cerebellar ataxia, neuronopathy, and vestibular areflexia) [10, 15], and also that CC should even be included as a symptom of CANVAS itself [13]. It has been recommended that patients with CANVAS be followed up by a multidisciplinary team that includes not only a neurologist, but also a pulmonologist and a gastroenterologist [14]. However, to our knowledge, no pathological studies on respiratory tissue in CANVAS cases.

As is the case for other neurological diseases such as Holmes-Adie syndrome [16] and type 1B sensory neuropathy [17], the mechanism underlying CC in CANVAS is currently unknown. CC is hypothesized to possibly be caused by impaired sensory innervation of upper respiratory tract or esophageal C fibres, resulting in their hypersensitivity [10, 13, 18]. The complex neurological mechanism behind CC involves a reflex arc made up of a peripheral and a central circuit.

The peripheral circuit is made up of afferent nerves, which activate or inhibit the cough mechanism through polymodal receptors, and efferent nerves, which activate the muscles involved in coughing. Subtypes of the afferent sensory nerves, part of the vagus nerve (10th cranial nerve), are the C fibres and A-delta fibres. C fibres are unmyelinated, have their cell bodies in the jugular ganglion, and are responsible for activating and inhibiting the cough reflex, while A-delta fibres are myelinated, have their cell bodies in the nodose ganglion, and are mostly responsible for activating the cough reflex [10, 19–21]. While peripheral terminations are believed to reside predominantly in the major airways (larynx, trachea, and large bronchi), terminations in the lung parenchyma cannot be ruled out, as several parenchymal lung diseases present with chronic cough [22]. Noxious stimuli, e.g., gastric fluid and cigarette smoke, are detected via receptors and ion channels (e.g., TRPV1 and P2X3) located in airway vagal afferent nerve endings [23]. Most C fibres are activated by irritant stimuli of inflammatory origin, while A-delta fibres are typically activated by mechanical and acidic stimuli [24]. As for the central circuit, this is made up of different regions such as the sensory cortical areas and the cingulate, insular, and orbitofrontal cortex [22]. Cough hypersensitivity, i.e., a coughing response to stimuli that do not usually produce cough, may be due to both peripheral and central mechanisms.

In the case of CANVAS, it is thought that AAGGGexp in intron 2 of *RFC1* may cause CC by damaging vulnerable tissues like the C and A-delta fibres [21]. This nerve fibre damage may provoke an early change in esophageal motility that may lead to gastroesophageal reflux (GER) and consequently may cause CC—a hypothesis that may be supported by several studies that document GER in patients with CANVAS [10, 13, 14, 25].

Bearing in mind the possible link between CC and CANVAS, to our knowledge no protocolized study applying European Respiratory Society (ERS) recommendations has been conducted by a multidisciplinary team that includes pulmonologists specializing in CC that has evaluated possible alterations and causes associated with CC and the impact on quality of life (QoL) in patients with CANVAS. Regarding a possible relationship between CT and CANVAS, Guilleminault et al*.* [26], in a recent study of the prevalence of repeat expansions of *RFC1* in patients with refractory CT, found that 16.2% (*n* = 11) and 8.8% (*n* = 6) of their patients had a biallelic mutation and a monoallelic mutation in *RFC1*, respectively.

Our aim was, as a multidisciplinary team composed of neurologists, pulmonologists, gastroenterologists, and geneticists, to identify clinical and functional characteristics of CC and its impact on QoL in patients with CANVAS.

## Materials and methods

### Study design and population

Our cross-sectional descriptive study included 10 male and female patients with CANVAS and experiencing CC, defined as a cough lasting > 8 weeks. The patients were referred to a specialist CC clinic from the neuromuscular disease unit of a Spanish tertiary hospital where they were being followed up for CANVAS, defined as the presence of the classical triad of cerebellar ataxia, neuronopathy, and vestibular areflexia, genetically confirmed by the biallelic expansion of AAGGG in *RFC1*. Exclusion criteria were age < 30 years and > 90 years, tobacco use, and angiotensin-converting enzyme inhibitor (ACEI) use, except when smoking was given up or ACEI use was withdrawn, respectively, and provided CC persisted.

## Methodology

Sociodemographic, clinical, and functional variables were collected for the 10 patients included in our CC study, which applied the ERS [23] protocol: chest X-ray and/or chest computed tomography (CT), spirometry with bronchodilator test (BDT), fraction of exhaled nitric oxide (FENO), prick test, total blood immunoglobulin E (IgE) count, absolute peripheral eosinophil count, 24-h esophageal pH test (24-h pH), and high-resolution esophageal manometry (HREM). CC impact was assessed using the following instruments: Cough Severity Diary (CSD) [27], Leicester Cough Questionnaire (LCQ) [28, 29], Discrete Emotions Questionnaire (DEQ) [30], and European Quality of Life-5 Dimensions-5 Levels (EQ-5D-5L) [31–33].

Spirometry with BDT was performed using the Datospir-600 device (Sibelmed SA, Barcelona, Spain) by an experienced technician and following American Thoracic Society/ERS (ATS/ERS) recommendations [34]. FENO was measured, applying ATS/ERS recommendations [35] and reference values from the literature [36], using electrochemical equipment (N-6008® SIR chemiluminescence sensor, Madrid, Spain) with precision ± 1%, a reading range of 0–500 parts per billion (ppb), and automatic results analysis. The prick test, based on the most prevalent standardized allergen extracts for our setting [37], was considered positive when the resulting papule was > 3 mm. Total blood IgE, determined using the enzyme-linked immunosorbent assay (ELISA) (UNICAP, Pharmacia, Uppsala, Sweden), was considered elevated for values > 160 IU/mL. 24-h pH was measured with a Digitrapper™ Recorder (Medtronic, Minneapolis, USA), using a 1-electrode probe placed 5 cm from the upper margin of the lower esophageal sphincter (LES) or a pH-impedance testing probe (pH-Z); the data were analysed using Reflux Software™ version 6.1 (Medtronic, Minneapolis, USA), and results were interpreted following Lyon Consensus recommendations [38]. Data for HREM, performed with the ManoScan™ system (Medtronic, Minneapolis, USA), were analysed using ManoView™ ESO version 3.3 software (Medtronic, Minneapolis, USA), and the findings were interpreted according to the Chicago Classification version 4.0 [39].

### Genetic study of biallelic AAGGG expansion in replication factor C subunit 1 (*RFC1*)

The patients’ DNA samples were analysed using 2 genetic techniques: (1) amplification by standard polymerase chain reaction (PCR) with primers flanking the intron 2 fragment of the *RFC1* gene (flanking-PCR)—a technique that only allows alleles of normal size to be amplified (i.e., not those containing large expansions, irrespective of their composition); and (2) amplification by triplet repeat primed PCR in 3 independent reactions: RP-PCR1 to detect the (AAAAG)11 and (AAAAG)exp alleles; RP-PCR2 to detect the (AAAGG)exp allele; and RP-PCR3 to detect the pathological (AAGGG)exp allele. The reference sequence used was NM_002913.4.

### Chronic cough-related quality of life in patients with CANVAS

CC-related QoL was investigated via responses to 3 hard-copy self-administered questionnaires, as follows:

1. CSD [27], designed to quantify cough severity in the previous 24 h, consists of 7 questions assessing frequency, intensity, and disruption, responded to on a Likert scale (0 = best possible score, 10 = worst possible score). The total score is obtained by summing and averaging the scores for the 3 domains, resulting in maximum and minimum possible scores of 10 and 0, respectively.
2. LCQ [28, 29], designed to assess the impact of coughing in the physical, psychological, and social domains in the previous 2 weeks, consists of 19 questions, responded to on a Likert scale (1 = worst possible score, 7 = best possible score). The total score is obtained by summing the scores for the 3 domains, resulting in maximum and minimum possible scores of 3 and 21, respectively.
3. DEQ [30], created to determine the impact of coughing on 32 emotions classified in 8 different emotional states (anger, disgust, fear, anxiety, sadness, desire, relaxation, and happiness). Each item is scored separately on a Likert scale (1 = worst possible score, 7 = best possible score).

In addition to these cough-specific questionnaires, health-related QoL was assessed using the EQ-5D-5L, which consists of 2 parts. For the first part, containing 5 health status questions with 5 responses reflecting different levels of severity, a specific reference value is subtracted depending on the target population (in our case, the Spanish population) [32]. Scores range from < 0 to 1, where 0 is equivalent to death, negative values reflect a status perceived to be worse than death, and 1 reflects full health [33]. The second part consists of a visual analogue scale (VAS), where 0 and 100 reflect the worst and best imaginable health states, respectively [31].

### Ethical and legal aspects

The study complied with the principles of the Declaration of Helsinki (18th World Medical Assembly, 1964) and was approved by the Hospital Santa Creu i Sant Pau Clinical Research Ethics Committee (Barcelona). Patients provided their informed written consent prior to participation in the study and all study data were anonymized. The clinicaltrials.gov identifier is NCT04703595.

## Results

### Patient clinical and functional characteristics

The CC study was performed in 10 patients with CANVAS (5 men and 5 women) who met all the inclusion criteria and none of the exclusion criteria. Mean age was 64.8 ± 8.6 years and mean body mass index (BMI) was 24.4 ± 3.2 kg/m^2^. The most frequent comorbidities were clinical GER (70%, *n* = 7), rhinitis (50%, *n* = 5), depression (50%, *n* = 5), and nocturnal sleep apnea (confirmed and undergoing study due to clinical suspicion in 40% (*n* = 4) and 30% (*n* = 3), respectively).

All patients reported dry-type CC (100%), with a mean duration of 30.4 ± 8.7 years, a mean onset of 34.4 ± 9.9 years previously, and a mean of 14.2 ± 7.9 years before the onset of neurological symptoms consistent with CANVAS. Coughing tended to worsen with strong odours (60%, *n* = 6), speech (60%, *n* = 6), eating (50%, *n* = 5), and stress (50%, *n* = 5). Dyspnea (50%, *n* = 5) and urinary incontinence (40%, *n* = 4) were also frequently associated with coughing attacks.

In most patients (70%, *n* = 7), CC had been studied prior to the CANVAS diagnosis by pulmonologists (40%, *n* = 4), otorhinolaryngologists (30%, *n* = 3), or gastroenterologists (30%, *n* = 3). However, no cause was identified for 4 of the patients, and treatment failed for 3 patients for whom a possible cause was identified. Follow-up was lost in 71.4% (*n* = 5) of those CC cases.

### Possible causes of chronic cough

Table 1 reports the full CC study results for the 10 patients with CANVAS. A single patient (#10) presented significant radiological and spirometry abnormalities, leading to a diagnosis of pleuro-parenchymal fibroelastosis. Notable at the lung function level was that 2 patients (#2 and #3) with spirometry in the normal range had a positive BDT. Only patient #3 showed partial improvement in response to the BDT, and this same patient also had associated peripheral eosinophilia and a positive prick test for dust mites and pollens, all compatible with a diagnosis of asthma.

**Table 1:** Patients with CANVAS: complementary CC test results

Case	Sex	Age	Chest x-ray	Chest CT	Spirometry	BDT	FENO (ppb)	Prick test (aeroallergens)	Eosinophils (× 10^9^/L) RV 0.10–0.50	IgE (kIU/L) RV 0.0–160.0	24-h pH	HREM
#1	M	73	No alterations of note	Chronic bronchopathy /mild lung emphysema	FEV1/FVC (%) 70.6 FEV1 2.33L 86% FVC 3.30L 93%	Negative	N/A	Negative	0.17	28.1	N/A	N/A
#2	F	73	No alterations of note	Slight esophageal distension	FEV1/FVC (%) 69.9 FEV1 1.74L 89% FVC 2.50L 102%	Positive	N/A	Negative	0.90	29.7	Pathological GER (non-cough related)	Ineffective esophageal motility/ no contraction reserve
#3	M	65	No alterations of note	No alterations of note	FEV1/FVC (%) 82.1 FEV1 2.50L 102% FVC 3.04L 98%	Positive	N/A	Dust mites/ pollen	0.56	142	Non-acid GER	Ineffective esophageal motility/ no contraction reserve
#4	F	67	N/A	No alterations of note	FEV1/FVC (%) 72 FEV1 2.46L 93% FVC 3.17L 101%	Negative	10	Negative	0.11	N/A	Pathological GER (non-cough related)	No esophageal motility/ no contraction reserve
#5	M	66	No alterations of note	Left-lung organizing pneumonia	FEV1/FVC (%) 78.4 FEV1 3.31L 98% FVC 4.26L 102%	Negative	N/A	Negative	0.60	N/A	N/A	No esophageal peristalsis/ LES hypotonia
#6	F	47	N/A	LLL polylobate ground-glass opacity	FEV1/FVC (%) 78.4 FEV1 2.58L 91% FVC 3.27L 93%	Negative	4	Dust mites	0.40	N/A	Not evaluable	UES hypertonia/small hiatal hernia
#7	F	52	No alterations of note	No alterations of note	FEV1/FVC (%) 71.8 FEV1 2.93L 106% FVC 4.08L 115%	Negative	N/A	Negative	0.80	N/A	Pathological non-acid GER	≥ 50% ineffective waves/ UES hypertonia with diminished relaxation
#8	F	70	Apical pleural calcifications	Apical pleural sequelae probably from past TB	FEV1/FVC (%) 86.3 FEV1 2.39L 123% FVC 2.77L 103%	Negative	67	Dust mites	0.83	54	Pathological GER (non-cough related)	Obstructed esophagogastric junction outflow
#9	M	66	Hyperinflation signs	Inflammatory small airway disease and air trapping signs	FEV1/FVC (%) 64.8 FEV1 2.49L 87% FVC 3.85L 99%	Negative	N/A	Negative	0.20	N/A	N/A	Ineffective esophageal motility/ no contraction reserve/ small hiatal hernia
#10	M	69	Calcified granulomas	Apical pleural thickening associated with fibrotic alterations	FEV1/FVC (%) 77.1 FEV1 2.02L 68% FVC 2.62L 65%	Negative	N/A	Negative	0.11	31.8	Pathological non-acid GER	Non-altered lung function

24-h pH, 24-h esophageal pH test; BDT, bronchodilator test; CANVAS, cerebellar ataxia with neuropathy and bilateral vestibular areflexia syndrome; CC, chronic cough; CT, computed tomography; F, female; FENO, exhaled fraction of nitric oxide; FEV1, forced expiratory volume in the first second; FVC, forced vital capacity; GER, gastroesophageal reflux; HREM, high-resolution esophageal manometry; IgE, immunoglobulin E; kIU/L, kilo international units per litre; LES, lower esophageal sphincter; LLL, left lower lobe; M, male; N/A, not available; RV, reference value; TB, tuberculosis; UES, upper esophageal sphincter

Over half the patients (#2, #3, #4, #5, #7, and #9) showed some degree of esophageal motility impairment, ranging from ≥ 50% of ineffective sequences (patient #7) to failed sequences (patient #4). The most frequent alteration was ineffective esophageal motility without contraction reserve (patients #2, #3, and #9). In patients #2, #4, and #8, 24-h pH testing indicated pathological GER, although none of these patients indicated that the GER was related to coughing symptoms during testing.

### Chronic cough impact on quality of life

Table 2 reports results for CC impact on QoL in the 10 patients with CANVAS. The mean total CSD score was 3.3 ± 1.9, and the impact was greater for the frequency (3.47 ± 2.2) and intensity (2.85 ± 1.9) domains than for the disruption domain. The mean total LCQ score was 13.1 ± 0.2, with a greater impact in the psychological domain (3.6 ± 1.1), followed by the social domain (4.4 ± 1.5) and the physical domain (5.1 ± 1.3). The DEQ domains most affected by CC were anxiety (3.2 ± 1.6) and anger (2.9 ± 1.8). As for the EQ-5D-5L, the mean health-related score was 0.8 ± 0.1 and the mean VAS score was 62 ± 12.5.

**Table 2:** Patients with CANVAS: questionnaire results for CC impact on quality of life

Cough severity diary (CSD)		Discrete Emotions Questionnaire (DEQ)	
*Domain*	*Score*	*Domain*	*Score*
Frequency	3.47 (± 2.2)	Anger	2.9 (± 1.8)
Intensity	2.85 (± 1.9)	Disgust	1.7 (± 1.0)
Disruption	1.85 (± 1.6)	Fear	2.5 (± 1.7)
Total	3.3 (± 1.9)	Anxiety	3.2 (± 1.6)
Leicester Cough Questionnaire (LCQ)		Sadness	2.2 (± 1.7)
*Domain*	*Score*	Desire	1.7 (± 0.7)
Physical	5.1 (± 1.3)	Relaxation	2 (± 1.1)
Psychological	3.6 (± 1.1)	Happiness	1.7 (± 1.9)
Social	4.4 (± 1.5)	*EQ-5D-5L*	
*Total*	13.1 (± .2)	VAS	62 (± 12.5)
		Health status	0.8 (± 0.1)
Better-to-worse scores: CSD = 1–10; LCQ = 7–1 (total = 21–3); DEQ = 1–7; EQ-5D-5L VAS = 100–0 and EQ-5D-5L health status = 1- < 0			

CANVAS, cerebellar ataxia with neuropathy and bilateral vestibular areflexia syndrome; CC, chronic cough; EQ-5D-5L, European Quality of Life-5 Dimensions-5 Levels; VAS, visual analogue scale

## Discussion

The main characteristics of CC in our patients with genetically confirmed CANVAS were that the cough was dry, appeared several years before the neurological symptoms of CANVAS, especially affected psychosocial QoL, and was mainly associated with pathological GER and esophageal motility abnormalities.

The fact that the CC was dry is a finding consistent with previous studies reporting dry spasmodic cough in a high percentage (50–60%) of patients with CANVAS [8, 12, 14, 39]. It has also been documented that coughing worsens in response to stimuli that do not normally produce cough, such as speech and stress [12, 13]. This worsening of the cough in response to stimuli that should not provoke cough may be compatible with cough hypersensitivity syndrome, a hypothesized cause of CC in patients with CANVAS that results from impaired nervous regulation of the cough reflex arc caused by innocuous irritants [10, 13, 18].

The questionnaires pointed to a negative impact of CC on the QoL of our patients with CANVAS, corroborating previous studies reporting an association between CC and impaired QoL [41–47]; note, however, that to date, the specific impact on patients with CANVAS has not been documented. The fact that the psychosocial domain was most affected by CC (4.4 ± 1.5 and 5.1 ± 1.3 for the LCQ psychological and social domains, respectively) corroborates a prospective study by French et al. [41] performed on 39 patients with CC of any etiology. Anxiety and anger were the most prevalent emotions (3.2 ± 1.6 and 2.9 ± 1.8 for DEQ anxiety and anger, respectively), corroborating a study by Ueda et al*.* [44] of 21 patients with refractory or unexplained CC. The fact that health-related QoL in our patients was also diminished by CC (EQ-5D-5L 0.8 ± 0.1) largely corroborates the cross-sectional studies by Won et al. [45], based on analysis of the Korean National Health and Nutrition Examination Survey 2010–2016 database, and by Kubo et al*.* [46], based on surveys of 1136 patients, half with CC and the other half without CC.

Regarding the complementary tests, notable were the findings of compatibility with asthma (20%) and GER (50%), corroborating results for other series of patients [10, 11, 13, 14]. However, treatment did not completely resolve coughing in our patients, while pathological GER episodes detected by 24-h pH testing were not related to cough but to episodes of heartburn. The fact that the CC remained unresolved despite suitable study and targeted treatment would suggest that refractory CC is a characteristic and persistent pneumological symptom in patients with CANVAS. For this reason, it is important to study CC in all patients with CANVAS, since targeted treatment may reduce intensity and frequency, and may even throw light on future targeted treatments for refractory CC.

As a possible cause of CC, another pulmonary disease, namely, pleuro-parenchymal fibroelastosis, was detected by radiology in a single patient (10% of the included patients) and required specific respiratory monitoring. This finding would suggest that patients with CANVAS require a multidisciplinary approach that includes a protocolized CC study based on assessment by a CC specialist from within the pulmonology discipline.

The fact that CC developed, on average, 14.2 ± 7.9 years before the onset of any CANVAS symptom in our patients highlights the importance of early diagnosis of this progressive and disabling neurological disease, suggesting that CANVAS, as an hereditary autosomal recessive disease, would benefit from early genetic counselling. CC should also be properly included as a symptom of CANVAS [13], rather than only be taken into account when associated with 2 of the triad of symptoms (cerebellar ataxia, neuronopathy, and vestibular areflexia) [10, 15].

A study strength is, as far as we are aware, that this is the first multidisciplinary study of patients with CANVAS that has involved neurologists, geneticists, and gastroenterologists, as well as pulmonologists and CC specialists. An additional strength is that the study applied current ERS recommendations. The main limitation is the small sample of just 10 patients, explained by both the low prevalence of CANVAS and the lack of neurological tests to determine CC pathophysiology in patients with CANVAS and to study the cough reflex arc. Precise data on CANVAS prevalence are unavailable, although prevalence is estimated to be < 1/1,000,000 in the general population [48].

## Conclusion

Although further studies are required for confirmation, CC onset up to decades before the development of neurological symptoms points to CC as a key early diagnostic clue for CANVAS. The importance of a multidisciplinary CC study of patients with genetically confirmed CANVAS is underlined by the fact that these patients present with a dry cough several years before the emergence of neurological symptoms, and that, in most patients, this cough, which especially affects psychosocial QoL, appears to be related to pathological GER and impaired esophageal motility. A multidisciplinary team of specialists is also necessary to confirm or rule out possible alternative causes of CC (such as asthma, GER, or pulmonary fibrosis) and to develop and provide targeted treatment for CC.
